# Ghrelin Therapy Improves Survival after Whole-Body Ionizing Irradiation or Combined with Burn or Wound: Amelioration of Leukocytopenia, Thrombocytopenia, Splenomegaly, and Bone Marrow Injury

**DOI:** 10.1155/2014/215858

**Published:** 2014-10-13

**Authors:** Juliann G. Kiang, Min Zhai, Pei-Jyun Liao, Thomas B. Elliott, Nikolai V. Gorbunov

**Affiliations:** ^1^Radiation Combined Injury Program, Armed Forces Radiobiology Research Institute, Bethesda, MD 20889, USA; ^2^Department of Radiation Biology, Uniformed Services University of the Health Sciences, Bethesda, MD 20814, USA; ^3^Department of Medicine, Uniformed Services University of the Health Sciences, Bethesda, MD 20814, USA

## Abstract

Exposure to ionizing radiation alone (RI) or combined with traumatic tissue injury (CI) is a crucial life-threatening factor in nuclear and radiological events. In our laboratory, mice exposed to ^60^Co-*γ*-photon radiation (9.5 Gy, 0.4 Gy/min, bilateral) followed by 15% total-body-surface-area skin wounds (R-W CI) or burns (R-B CI) experienced an increment of ≥18% higher mortality over a 30-day observation period compared to RI alone. CI was accompanied by severe leukocytopenia, thrombocytopenia, erythropenia, and anemia. At the 30th day after injury, numbers of WBC and platelets still remained very low in surviving RI and CI mice. In contrast, their RBC, hemoglobin, and hematocrit were recovered towards preirradiation levels. Only RI induced splenomegaly. RI and CI resulted in bone-marrow cell depletion. In R-W CI mice, ghrelin (a hunger-stimulating peptide) therapy increased survival, mitigated body-weight loss, accelerated wound healing, and increased hematocrit. In R-B CI mice, ghrelin therapy increased survival and numbers of neutrophils, lymphocytes, and platelets and ameliorated bone-marrow cell depletion. In RI mice, this treatment increased survival, hemoglobin, and hematocrit and inhibited splenomegaly. Our novel results are the first to suggest that ghrelin therapy effectively improved survival by mitigating CI-induced leukocytopenia, thrombocytopenia, and bone-marrow injury or the RI-induced decreased hemoglobin and hematocrit.

## 1. Introduction

Large-scale radiation exposure events in history have shown that irradiated victims are also often subjected to other trauma such as wounds or burns. Combined injuries (CIs) were observed at Hiroshima and Nagasaki, Japan, where 60–70% of victims received thermal burns concurrent with radiation injury [1, 2]. At the Chernobyl reactor meltdown, 10% of 237 victims exposed to radiation received thermal burns as well [3]. In animal models of CI including mice [4–12], rats [13–15], guinea pigs [16], dogs [17, 18], and swine [19], burns, wounds, and infections usually increase mortality after otherwise nonlethal radiation doses. In rodents, radiation combined with burns, wounds, or infections decreases survival compared to radiation alone [4–12]. Radiation injury (RI) also delays wound closure times [4, 7, 8, 20].

Consequences of CI include expedited body-weight loss, magnified cytokine and chemokine imbalance, systemic bacterial infection [4], enhanced leukocytopenia, thrombocytopenia, and erythropenia [5, 7, 8], acute myelosuppression, immune system inhibition, fluid imbalance, macro- and microcirculation failure, massive cellular damage, and disruption of vital organ functions, which, as is the case with radiation exposure alone, can lead to multiple organ dysfunction and multiple organ failure, the most frequent causes of death after CI [16, 21, 22]. The LD_50/30_ for radiation-wound CI (R-W CI) is 8.95 Gy, while the LD_50/30_ for RI is 9.65 Gy. The dose modifying factor (DMF) is 1.08 [4]. We hypothesized that intervention addressing CI-induced enhancements of body-weight loss, cytokine imbalance, or systemic bacterial infection would improve the survival (see review, [23]).

Ghrelin is a hunger-stimulating peptide and hormone containing 28 amino acids [24]. Inui et al. [25] reported that ghrelin is produced mainly by P/D1 cells lining the fundus of the human stomach and epsilon cells of the pancreas. Ghrelin levels increase before meals and decrease after meals. It is considered a counterpart to the hormone leptin, produced by adipose tissue [26].

It was reported that ghrelin is a potent stimulator of growth hormone from the anterior pituitary gland [24]. The ghrelin receptor is a G protein-coupled receptor, known as the growth hormone secretagogue receptor that is coupled to G-protein. Ghrelin was shown to activate the endothelial isoform of nitric oxide synthase (eNOS) in a pathway [27] that depended on various kinases including PI3K/Akt/eNOS/NO signal pathway [28, 29].

Shah et al. [14] reported that human ghrelin treatment significantly reduced organ injury and improved survival by 30% above the vehicle-treated mice after RI combined with severe sepsis in rats. Whether ghrelin therapy would be efficacious in a R-W or R-B CI model was unknown. Peng et al. [30] reported that ghrelin inhibited NF-*κ*B activation, reduced TNF-*α* and IL-6 concentrations in lung of septic rats, and inhibited nucleotide-binding oligomerization domain-containing protein 2 (NOD2), also known as caspase recruitment domain-containing protein 15 (CARD15) or inflammatory bowel disease protein 1 (IBD1). NOD2 plays a role in apoptosis and the NF-*κ*B activation pathways [31]. Dixit et al. [32] showed that ghrelin inhibited I*κ*B and increased Th1 cytokine and IL-17 secretion in primary T cells. Further, it is well recognized that RI and R-W or R-B CI induce increased production of inflammatory cytokines and chemokines [4–6] and it is thought that interventions which would mitigate inflammatory responses could likely improve survival after R-W or R-B CI. We, therefore, hypothesized that ghrelin therapy could contribute to improved survival as well as mitigate other vital cellular and tissue parameters. To prove our hypothesis, this report provides data from an experimental CI animal model, which was designed to demonstrate the efficacy of ghrelin as an effective radiomitigator/therapy agent.

## 2. Materials and Methods

### 2.1. Experimental Design

B6D2F1/J female mice were randomly divided into 12 groups: (1) sham control, (2) wound control, (3) radiation (RI) control, (4) radiation followed by wound (CI) control, (5) sham vehicle, (6) wound vehicle, (7) RI vehicle, (8) CI vehicle, (9) sham ghrelin, (10) wound ghrelin, (11) RI ghrelin, and (12) CI ghrelin. Groups 6, 8, 10, and 12 received topical gentamicin cream; groups 5–12 were administered with oral levofloxacin. Survival experiments were repeated 1 time with *N* = 10-11 mice per group. Hematological analysis, spleen weights, splenocyte counts, and bone marrow cell counts of surviving animals were performed at the end of survival monitoring periods.

### 2.2. Animals

B6D2F1/J female mice (The Jackson Laboratory, Bar Harbor, ME) were maintained in a facility accredited by the Association for Assessment and Accreditation of Laboratory Animal Care International in plastic microisolator cages on hardwood chip bedding. Commercial rodent chow and acidified tap water were provided* ad libitum* at 12 to 20 weeks of age. Animal holding rooms were maintained at 21°C ± 1°C with 50% ± 10% relative humidity using at least 10 changes/h of 100% conditioned fresh air. A 12-h 0600 (light) to 1800 (dark) full-spectrum lighting cycle was used. The AFRRI Institutional Animal Care and Use Committee reviewed and approved all animal procedures. Euthanasia was carried out in accordance with the recommendations and guidance of the American Veterinary Medical Association [33, 34].

### 2.3. Gamma Irradiation

Mice were given 9.5 Gy [5] whole-body bilateral ^60^Co *γ*-photon radiation, delivered at a dose rate of 0.4 Gy/min, while held in vertically stacked, ventilated, four-compartment, acrylic plastic boxes that provided electron equilibrium during irradiation. Empty compartments within the boxes were filled with 3-inch-long × 1-inch-diameter acrylic phantoms to ensure uniform electron scattering. The mapping of the radiation field was performed with alanine/EPR dosimetry [35] using standard alanine calibration sets from US National Institute of Standards and Technology and National Physical Laboratory of the United Kingdom. The mapping provided dose rates to water measured by alanine pellets placed in the hollowed core of the acrylic phantoms in each compartment of the mouse rack on the day of the mapping. The field was uniform within ±1.8% over all of the 120 compartments. The exposure time for each irradiation was determined from the mapping data; corrections for the ^60^Co decay and the small difference in the mass energy absorption coefficients for water and soft tissue were applied. The accuracy of the actual dose delivery was verified with an ionization chamber adjacent to the mouse rack, which had been calibrated in terms of dose to the mid-line soft tissue of mice.

### 2.4. Skin Injury

Skin surface injuries were performed on the shaved dorsal surface of mice. Animals receiving skin burns were anesthetized by methoxyflurane inhalation. A 15% total-body-surface-area skin burn was performed within 1 h after irradiation using a 1 × 1-inch custom designed template positioned centrally over the shaved dorsal skin surface. A volume of 0.25 mL of 95% ethanol was applied evenly to the dorsal skin surface, which was exposed by the template. The ethanol was ignited and allowed to burn for 12 s [6, 9]. All mice subjected to the skin injury were given 0.5 mL sterile 0.9% NaCl intraperitoneally (i.p.), which contained 150 mg/kg of acetaminophen (AmerisourceBergen, Glen Alen, Virginia) and 0.05 mg/kg of buprenorphine immediately after skin injury to alleviate pain. Four hours later, mice were given a second dose of 150 mg/kg of acetaminophen. For animals receiving skin wounds, animals were anesthetized by isoflurane inhalation and a 15% total body-surface-area skin wound was performed within 1 h after irradiation [6, 9]. Skin-wounded mice received only one dose of 150 mg/kg of acetaminophen immediately after skin injury.

### 2.5. Ghrelin

Ghrelin was purchased from Phoenix Pharmaceutical (Burlingame, CA). Three doses of 113 *μ*g/kg were administered by lateral tail-vein injections [14] in a volume of 0.2 mL 24 h, 48 h, and 72 h after RI or CI. The vehicle given to control mice was sterile 0.9% sodium chloride solution for injection, USP.

### 2.6. Antimicrobial Agents

Gentamicin sulfate cream, 0.1% (generic, E. Fougera and Co., Melville, N.Y., NDC 0168-007-15), was applied daily for 10 days to the skin injuries on days 1–10. Levofloxacin (LVX), (generic, Aurobindo Pharma, Ltd., Mahaboob Nagar, India, NDC 65862-537-50), 100 mg/kg in 0.2 mL/mouse, was administered* p.o*. daily for 14 days on days 3–16. Briefly, a 500 mg tablet was crushed by mortar and pestle. The LVX in the powder was dissolved in a volume of sterile water approximately one-third of the total volume required to prepare the concentration needed for the average body mass of the mice to be treated. The mortar was rinsed with the remaining two-thirds volume of sterile water. The combined suspension was centrifuged to remove the particulate filler and the supernatant solution was passed through a 0.45 *μ*m membrane filter into a sterile amber bottle, which was sealed with a sterile rubber stopper and stored at 4–8°C [7].

### 2.7. Survival and Body Weight

Animals were monitored at least twice daily for their general health and survival for 30 days. Their body weights were measured on days 0, 1, 3, 7, 14, 21, and 28.

### 2.8. Assessment of Blood Cell Profile in Peripheral Blood

Blood samples were collected in EDTA tubes at day 30 after RI or CI and assessed with the ADVIA 2120 Hematology System (Siemens, Deerfield, IL). Differential analysis was conducted using the peroxidase method and the light scattering techniques recommended by the manufacturer.

### 2.9. Measurements of Spleen Weights and Splenocytes

Spleens were collected from each euthanized mouse at day 30 after RI or CI. Each specimen was weighed and then homogenized in a cell strainer (BD Falcon, Bedford, MA) with 1X Hank's Balanced Salt Solution (Invitrogen, Grand Island, NY). Splenocytes in the buffer were washed with 1X ACK lysis buffer (Invitrogen) to lyse RBC, mixed by vortexing, and centrifuged at 800 ×g. Splenocytes were collected and placed in Countess cell-counting-chamber slides (Invitrogen, Eugene, Oregon) and counted using Countess automated cell counter (Invitrogen).

### 2.10. Measurements of Bone Marrow Cells

Bone marrow cells from femurs were collected at day 30 after RI or CI and washed with 10 mL 1X phosphate-buffered saline (PBS). The cells were then centrifuged at 800 ×g and resuspended in 10 mL 1X PBS buffer and were then placed in Countess cell-counting-chamber slides (Invitrogen) and counted using Countess automated cell counter (Invitrogen).

### 2.11. Statistical Analysis

Parametric data are expressed as the mean ± s.e.m. For each survival experiment, 20–22 mice per group were tested on an individual basis. Survival analyses were performed using the log-rank test. For cell analysis, one-way ANOVA, two-way ANOVA, studentized-range test, and Student's *t*-test were used for comparison of groups, with 5% as a significant level.

## 3. Results

### 3.1. Survival and Body Weight

**Figure 1:**
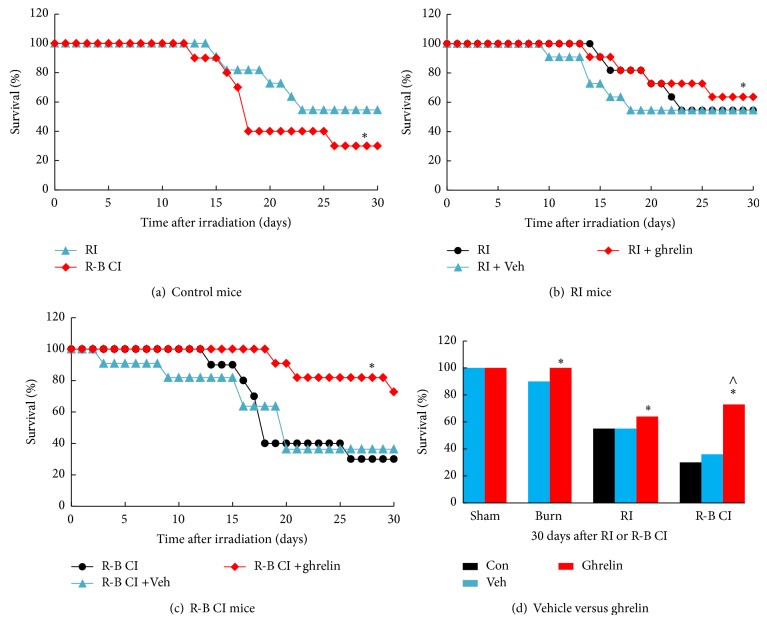
Ghrelin improved survival after whole-body ionizing irradiation combined with skin burn. *N* = 10-11 per group; experiment was repeated once. For panel (a): ^*^ *P* < 0.05 versus RI. For panel (b): ^*^ *P* < 0.05 versus RI and RI + Veh. For panel (c): ^*^ *P* < 0.05 versus R-B CI and R-B CI + Veh. For panel (d): representing 73% versus 36% survival in ghrelin-treated and vehicle-treated R-B CI mice, respectively. ^*^ *P* < 0.05 versus RI + Veh; ^∧^ *P* < 0.05 versus RI + Ghrelin. Veh: vehicle; RI: 9.5 Gy; R-B CI: 9.5 Gy and skin burn.

Ghrelin was tested in an R-B CI mouse model. Irradiation was followed within 1 h by dorsal skin burns. Mice were divided into 12 treatment groups in a 3 × 4 design: mice (*n* = 10 or 11 per treatment group, repeated once), which were given sham irradiation, skin burn only, radiation only (RI), and combined injury (R-B CI), were given either ghrelin, vehicle, or sham treatment only. Skin burn following irradiation increased mortality to 70%, which was greater than mortality observed in RI mice (45%; *P* < 0.05), as shown in Figure 1(a). In RI mice, vehicle treatment did not affect the radiation-induced mortality (Figures 1(b) and 1(d)). Treatment with ghrelin, however, enhanced 30-day survival from 55% to 64% (Figure 1(b); *P* < 0.05). In R-B CI mice, ghrelin treatment increased survival from 36% to 73% after R-B CI (Figures 1(c) and 1(d); *P* < 0.05). Skin burn (15% total-body-surface area) alone resulted in 5% mortality over a 30-day observation period [7].

**Figure 2:**
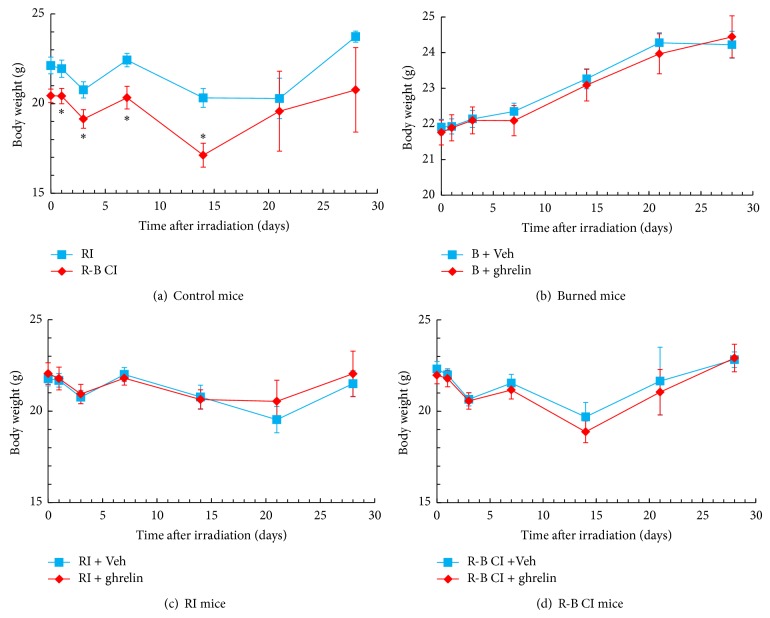
Ghrelin did not improve body-weight loss after RI or R-B CI. *N* = 10-11 per group; experiment was repeated once. ^*^ *P* < 0.05 versus RI. RI: 9.5 Gy; R-B CI: 9.5 Gy and skin burn.

Skin burn enhanced the radiation-induced body-weight loss (Figure 2(a)) but skin burn alone did not induce body-weight loss (Figure 2(b)). RI is known to decrease the body weight of mice [5]. Ghrelin treatment did not reduce the body-weight loss in the RI mice (Figure 2(c)) or the R-B CI mice (Figure 2(d)).

**Figure 3:**
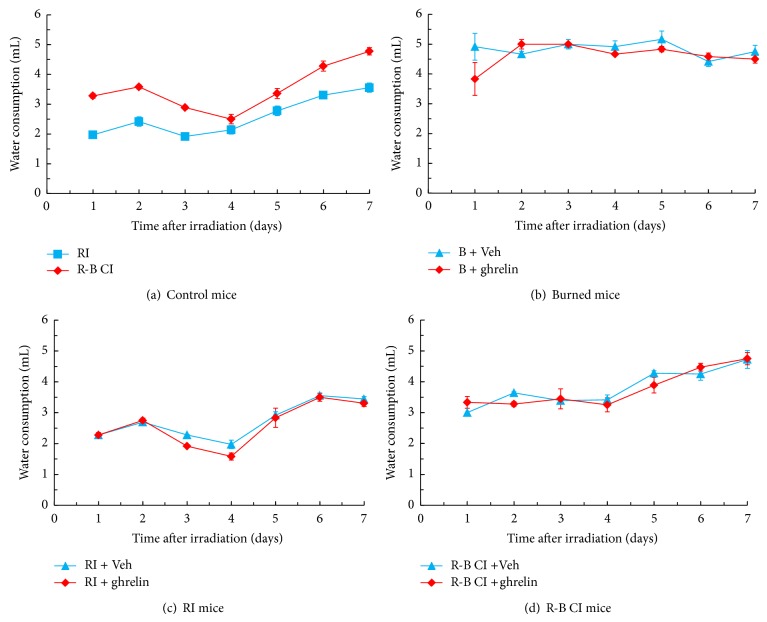
Ghrelin did not modify water consumption after RI or R-B CI. *N* = 10-11 per group; experiment was repeated once. Each vehicle-treated nonirradiated mouse normally drank 3.69 ± 0.17 mL/day. B: burn; RI: 9.5 Gy; R-B CI: 9.5 Gy and skin burn.

Ghrelin treatment did not alter water consumption compared to vehicle-treated mice (Figure 3). Each vehicle-treated nonirradiated mouse normally drank 3.69 ± 0.17 mL/day (*n* = 10, repeated once). RI has been shown to suppress water consumption, whereas R-B CI stimulates water consumption due to the water evaporation through the burned area [36] compared to RI mice but normal consumption resumed by day 7 after RI or R-B CI [5].

**Figure 4:**
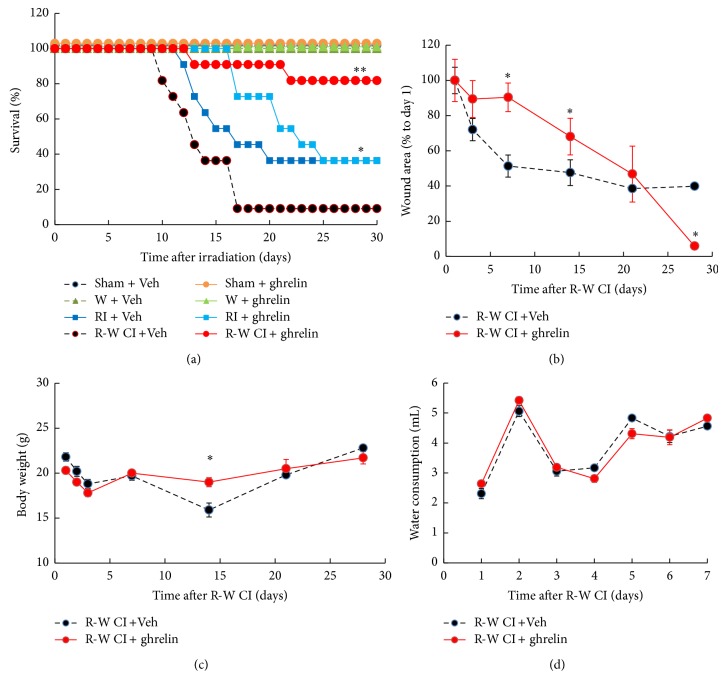
Ghrelin improved survival, body weight, and wound healing after whole-body ionizing irradiation combined with skin wound. *N* = 10-11 per group; experiment was repeated once. For panel (a): survival ^*^ *P* < 0.05 versus R-W CI + Veh; ^**^ *P* < 0.05 versus RI + Veh, RI + Ghrelin, and R-W CI + Veh. For panel (b): wound closure ^*^ *P* < 0.05 versus R-W CI + Veh. For panel (c): body weight, ^*^ *P* < 0.05 versus R-W CI + Veh. For panel (d): each vehicle-treated nonirradiated mouse normally drank 3.64 ± 0.19 mL/day. Water consumption between vehicle and ghrelin treatments showed no difference. Veh: vehicle; RI: 9.5 Gy; R-W CI: 9.5 Gy and skin wound.

In a separate experimental protocol, skin wound was performed following irradiation in an experimental design similar to that described for the skin burn model. Ghrelin was administered* i.v.* daily on days 1–3. Survival in radiation-wound combined injured (R-W CI) mice given ghrelin was increased from 9% (vehicle-treated R-W CI mice) to 82% (ghrelin-treated R-W CI mice, *P* < 0.05) during the 30-day experimental period (Figure 4(a)). Although total survival of RI mice treated with ghrelin or vehicle was the same in this experiment, that is, 36%, ghrelin extended survival by five days, a period which allows the use of other interventions. All nonirradiated mice given ghrelin survived (Figure 4(a)). Ghrelin reduced body-weight loss by the 14th day in R-W CI mice (Figure 4(c)). In the ghrelin-treated R-W CI mice, the wound healed to a full closure by day 20, whereas in the vehicle-treated R-W CI mice the wound was not fully healed yet even by day 30 (Figure 4(b)). Each vehicle-treated nonirradiated mouse normally drank 3.64 ± 0.19 mL/day (*n* = 10, repeated once). RI mice drank less water than did sham-irradiated mice while the R-W CI mice drank more water due to water evaporation through the open wound [36]. However, the water consumption was not different between vehicle-treated and ghrelin-treated groups (Figure 4(d)).

### 3.2. Blood Cell Profile in Peripheral Blood

**Figure 5:**
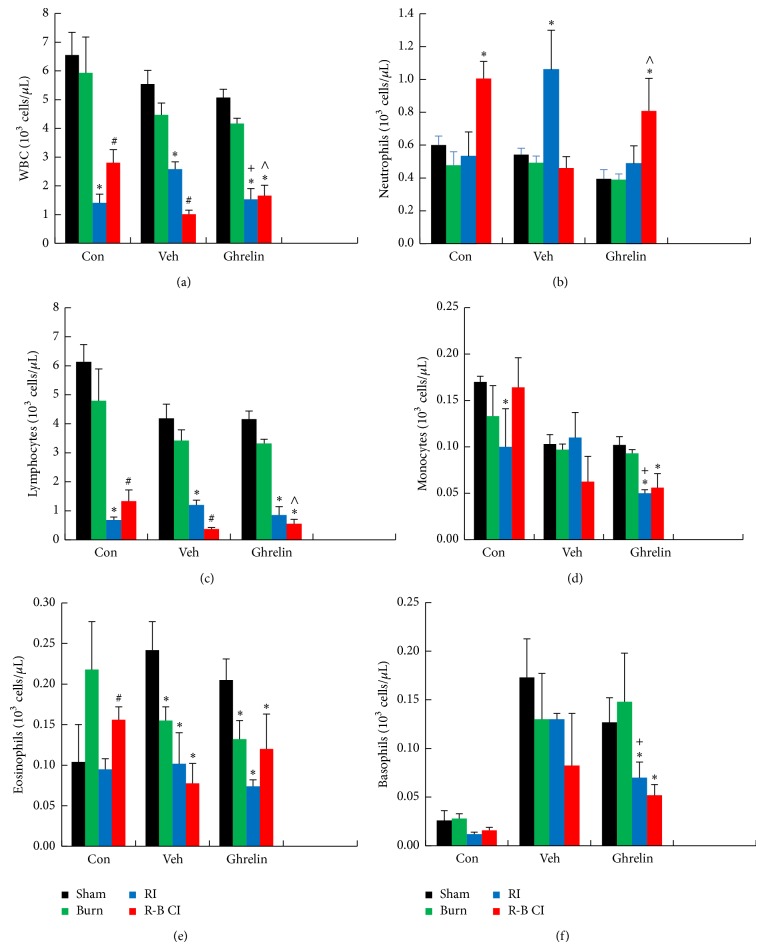
Ghrelin mitigated WBC depletion 30 d after irradiation combined with skin burn. *N* = 4–6 per group. ^*^ *P* < 0.05 versus respective sham; ^#^ *P* < 0.05 versus respective RI; ^+^ *P* < 0.05 versus RI + Veh; ^∧^ *P* < 0.05 versus CI + Veh. Con: control; Veh: vehicle; RI: 9.5 Gy; CI: 9.5 Gy and skin burn.

In surviving RI mice, ghrelin treatment aggravated WBC depletion compared to vehicle (Figure 5(a)), mainly in the numbers of neutrophils (Figure 5(b)), monocytes (Figure 5(d)), and basophils (Figure 5(f)) but not lymphocytes. In surviving R-B CI mice, ghrelin treatment significantly mitigated WBC depletion (Figure 5), mainly in numbers of neutrophils (Figure 5(b)) and lymphocytes (Figure 5(c)). Skin burn alone did not significantly alter WBC counts.

**Figure 6:**
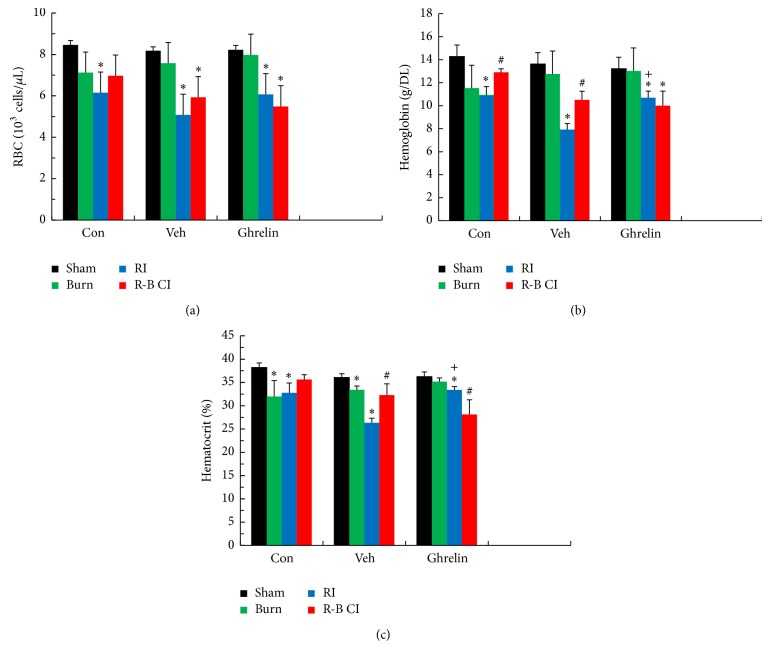
Ghrelin elevated hemoglobin levels and hematocrit readings 30 days after irradiation. *N* = 4–6 per group. ^*^ *P* < 0.05 versus respective sham; ^#^ *P* < 0.05 versus respective RI; ^+^ *P* < 0.05 versus RI + Veh. Con: control; Veh: vehicle; RI: 9.5 Gy; CI: 9.5 Gy and skin burn.

In surviving RI mice, ghrelin treatment did not alter RBC numbers significantly (Figure 6(a)) but increased hemoglobin (Figure 6(b)) and hematocrit (Figure 6(c)). In surviving R-B CI mice, ghrelin therapy did not alter these three parameters (Figures 6(a)–6(c)). In comparison, RI alone decreased RBC, hemoglobin, and hematocrit as was demonstrated previously [5, 7]. Skin burn alone decreased hematocrit but not RBC or hemoglobin (Figures 6(a)–6(c)).

**Figure 7:**
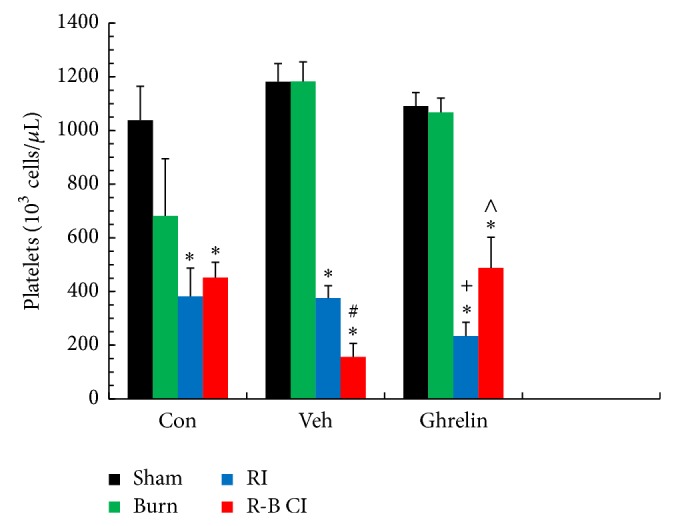
Ghrelin mitigated platelet depletion 30 days after irradiation combined with skin burn. *N* = 4–6 per group. ^*^ *P* < 0.05 versus respective sham; ^#^ *P* < 0.05 versus respective RI; ^+^ *P* < 0.05 versus RI + Veh; ^∧^ *P* < 0.05 versus CI + Veh. Con: control; Veh: vehicle; RI: 9.5 Gy; CI: 9.5 Gy and skin burn.

In vehicle-treated groups, RI significantly reduced numbers of platelets that were further reduced after R-B CI. Ghrelin therapy in RI mice decreased platelets more than in vehicle-treated mice, while the therapy significantly elevated platelets in R-B CI mice (Figure 7). The number of platelets was decreased in RI and R-B CI mice as demonstrated previously [5, 7].

### 3.3. Spleen Weight and Splenocytes

**Figure 8:**
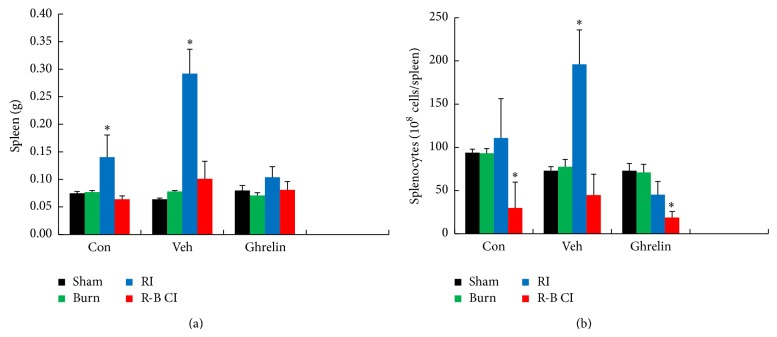
Ghrelin mitigated both increased spleen weights and decreased splenocyte counts 30 days after irradiation. *N* = 6 per group. ^*^ *P* < 0.05 versus respective sham and burn. Con: control; Veh: vehicle; RI: 9.5 Gy; CI: 9.5 Gy and skin burn.

RI alone significantly increased spleen weight (i.e., splenomegaly) as demonstrated only in surviving mice at 30 days, which indicated recovery, whereas R-B CI did not alter spleen weight but did decrease number of splenocytes. Skin burn alone did not alter spleen weights or the number of splenocytes (Figure 8). Ghrelin treatment fully inhibited radiation-induced increases in spleen weights (Figure 8(a)) and splenocyte counts (Figure 8(b)) in RI mice. Treatment with ghrelin did not alter spleen weights (Figure 8(a)) but reduced further already decreased splenocyte counts in R-B CI mice (Figure 8(b)).

### 3.4. Bone Marrow Cells

**Figure 9:**
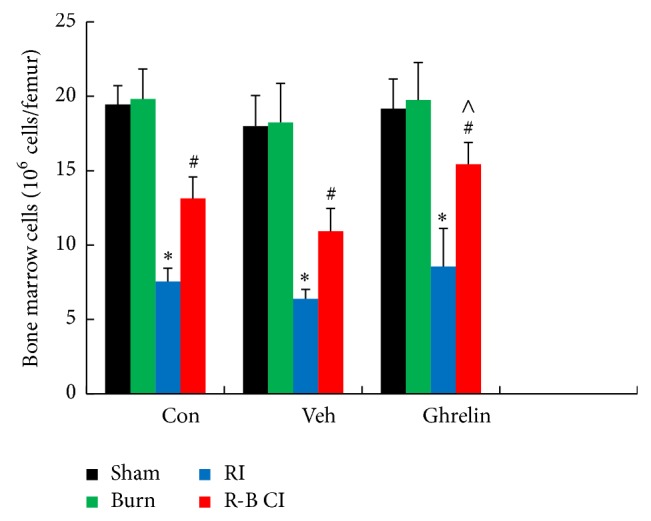
Ghrelin increased bone marrow cell counts 30 days after irradiation combined with skin burn. *N* = 6 per group. ^*^ *P* < 0.05 versus respective sham and burn; ^#^ *P* < 0.05 versus respective RI; ^∧^ *P* < 0.05 versus CI + Veh. Con: control; veh: vehicle; RI: 9.5 Gy; CI: 9.5 Gy and skin burn.

In RI mice, irradiation significantly decreased the bone marrow cell count in all treatment groups. Ghrelin treatment in RI mice decreased bone marrow cell counts even more than vehicle treatment in RI mice. In R-B CI mice, ghrelin treatment significantly improved the bone-marrow cellularity, which was higher than in vehicle-treated CI mice (Figure 9). Skin burn alone did not change the bone-marrow cellularity.

## 4. Discussion

Our novel results are the first to show that ghrelin enhanced survival in RI and CI mice, reduced body-weight loss, accelerated skin-wound healing, increased numbers of neutrophils, lymphocytes, and platelets, and elevated bone marrow cells after CI. Ghrelin therapy also increased hemoglobin levels and hematocrit readings as well as blockade of splenomegaly after RI. The results suggest that ghrelin is a promising candidate therapeutic agent, which could extend survival several days and contribute to a combination of interventions that would modulate the complex molecular responses to CI as well as RI. Although results of cellular parameters revealed differences in responses between RI and CI mice, the disparities raise the opportunity for further investigation to determine definitive explanations for the underlying reasons.

Ghrelin, a stomach-derived peptide, has a half-life of approximately 31 min in plasma [37, 38]. It was reported recently as a countermeasure against radiation combined with sepsis [14]. It was suggested that the effect was based on complex neurogenic effects of this peptide. Whole-body radiation combined with polymicrobial sepsis activated the sympathetic nervous system and led to the release of norepinephrine (NE) from the sympathetic fibers in the gut [14, 39, 40]. The NE then traveled through the portal vein into the liver. While in the liver, NE bound to 2A-adrenoceptors (2A-AR) and activated signaling pathway(s) responsible for the production and release of proinflammatory cytokines, TNF-*α*, IL-6, IL-1*β*, and HMGB-1 (high-mobility-group B1), from Kupffer cells. Evidence indicated that ghrelin, when it was released from the stomach, entered the dorsal vagal complex in the brain by crossing the blood-brain barrier and stimulated GHSR-1a (i.e., ghrelin receptors, coupling to G protein) and then activated the vagus nerve and, in turn, through the cholinergic pathways, downregulated TNF-*α* and other proinflammatory cytokines [39]. While activating the cholinergic pathway, ghrelin inhibited the sympathetic nervous system (SNS), thus decreasing the release of the sympathetic neurotransmitter, NE, and caused a downregulation of the proinflammatory cytokines as well (see reviews, [39, 41]). Ghrelin's beneficial effects following irradiation combined with sepsis may have been correlated with the rebalance of the dysregulated sympathetic and parasympathetic (PNS) nervous systems [39]. It is possible, therefore, that ghrelin-induced improvement of survival in our CI models is mediated by the rebalance of cytokines, SNS, and PNS. This hypothesis requires confirmation.

RI and CI significantly reduced WBC, RBC, and platelet counts as previously reported [5, 7]. In the current study, by day 30 after RI or R-B CI, surviving mice still displayed low values of WBC, mainly lymphocytes, monocytes, and eosinophils (Figure 5). However, in R-B CI mice, ghrelin therapy increased neutrophils and ameliorated loss of lymphocytes, suggesting that ghrelin accelerates bone-marrow cell proliferation and maturation, as confirmed in Figure 9. Ghrelin may have acted via GHSR-1*α*-mediated PI3K/Akt/eNOS/NO signal pathway [29] to initiate proliferation and differentiation of myeloid progenitors into mature granulocytes and induce hematopoietic stem-cell mobilization from the bone marrow into the bloodstream. These effects promote recovery from infection [42, 43] and wound healing [44]. Moreover, ghrelin is known to modulate immune responses [45]. Thus, it is possible that ghrelin's effectiveness in survival improvement is mediated by its ability to enhance recovery from infection, wound healing, and immunity after CI that are major issues leading to lethality. Because GHSR is expressed in lymphocytes, the action of ghrelin directly to preserve lymphocytes cannot be ruled out.

Ghrelin mitigated RI-induced decreases in hemoglobin and hematocrit levels. This mitigation may significantly contribute to the survival improvement after RI (Figure 1(c)). However, it is elusive why ghrelin did not mitigate the R-B CI-enhanced decreases in these three parameters. Nevertheless, ghrelin treatment improved numbers of platelets in R-B CI-mice surviving for 30 days but not in surviving RI-mice. This differential observation might suggest that this hormone also could stimulate megakaryocytes in the bone marrow under certain conditions, which are not defined here, similar to platelet recovery resulting from IL-12 treatment [46] and pegylated G-CSF [7]. From our results, we postulate that ghrelin mobilizes myeloid cells and megakaryocytes to peripheral blood to mitigate the blood-cell depletion in R-B CI surviving mice (Figures 5(b), 5(d), and 5(f), and Figure 7), a previously unrecognized effect of ghrelin.

We observed that the RI mice but not CI mice exhibited splenomegaly and that ghrelin mitigated the RI-induced splenomegaly. Splenomegaly is usually associated with disease processes that involve the destruction of abnormal RBC in the spleen. Splenomegaly may not only be caused by removal of RBC after irradiation but a possible overproduction of splenic T cells might also contribute to this effect. Ghrelin might inhibit proliferation of splenic T cells [47] so that splenomegaly is no longer present. Further studies to elucidate the mechanisms of RI-induced splenomegaly will be required to explain the different responses of RI and CI mice.

Ghrelin was found to reduce Bax (a proapoptotic protein) and increase Bcl-2 (an anti-apoptotic protein) in a chronic liver injury model [27]. Determining the differential activities of ghrelin on Bax and Bcl-2 in RI and CI mice would be informative, considering that mice in these models of acute injuries do not have chronic liver disease.

We hope this report enables to stimulate interest in advancing research on ghrelin in support of eventual approval for treatment of CI-induced leukocytopenia, thrombocytopenia, or bone marrow injury by the US Food and Drug Administration. To advance the ghrelin efficacy as a therapy, the optimal dosing and the easy implementation of administration routes (i.e., i.m. or s.c.) using autoinjectors shall be explored. Experiments for CIP dose reduction factor (DRF) shall be carried out and DRF shall be calculated and determined. Furthermore, testing out with larger animals such as minipigs or nonhuman primates shall be conducted.

In summary, skin burns or wounds increased radiation-induced mortality and body-weight loss. Ghrelin treatment enhanced 30-day survival after CI, significantly accelerated wound healing, mitigated body-weight loss, leukocytopenia, thrombocytopenia, and bone-marrow injury in CI mice. This treatment also increased survival, hemoglobin levels, and hematocrit readings and inhibited splenomegaly in RI-mice. These results demonstrate efficacy of ghrelin as a radiomitigator/therapy agent for CI and RI.

## Abbreviations

AFRRI: Armed Forces Radiobiology Research Institute
B: Burn
CI: Combined injury
GHSR: Growth hormone secretagogue receptor
i.m.: Intramuscular
i.p.: Intraperitoneal
i.v.: Intravenous
p.o.: Oral gavage
RBC: Red blood cells
RCI: Radiation combined injury
R-B CI: Radiation-burn combined injury
R-W CI: Radiation-wound combined injury
RI: Radiation injury
s.c.: Subcutaneous
TBSA: Total body surface area
VSD: Veterinary Science Department
W: Wound
WBC: White blood cells.

## Disclaimer

The views, opinions, and findings contained in this report are those of the authors and do not reflect official policy or positions of the Armed Forces Radiobiology Research Institute, the Uniformed Services University of the Health Sciences, the National Institute of Allergy and Infectious Diseases, the Department of Defense, or the United States government. The commercial products identified in this paper do not imply recommendation or endorsement by the federal government and do not imply that the products identified are necessarily the best available for the purpose.

## References

[1] Iijima S. (1982). Pathology of atomic bomb casualties. *Acta Pathologica Japonica*.

[2] Shimada Kishi H., Carey M. E. (2000). Effects of the “special bomb”: recollections of a neurosurgeon in Hiroshima, August 8–15, 1945. *Neurosurgery*.

[3] Barabanova A. V. (2006). Significance of beta-radiation skin burns in Chernobyl patients for the theory and practice of radiopathology. *Vojnosanitetski Pregled*.

[4] Kiang J. G., Jiao W., Cary L. H., Mog S. R., Elliott T. B., Pellmar T. C., Ledney G. D. (2010). Wound trauma increases radiation-induced mortality by activation of iNOS pathway and elevation of cytokine concentrations and bacterial infection. *Radiation Research*.

[5] Kiang J. G., Garrison B. R., Burns T. M., Zhai M., Dews I. C., Ney P. H., Cary L. H., Fukumoto R., Elliott T. B., Ledney G. D. (2012). Wound trauma alters ionizing radiation dose assessment. *Cell & Bioscience*.

[6] Kiang J. G., Ledney G. D. (2013). Skin inqjuries reduce survival and modulate corticosterone, C-reactive protein, complement component 3, IgM, and prostaglandin Eafter whole-body reactor-produced mixed field (n + *γ*-photons) irradiation. *Oxidative Medicine and Cellular Longevity*.

[7] Kiang J. G., Zhai M., Liao P.-J., Bolduc D. L., Elliott T. B., Gorbunov N. V. (2014). Pegylated G-CSF inhibits blood cell depletion, increases platelets, blocks splenomegaly, and improves survival after whole-body ionizing irradiation but not after irradiation combined with burn. *Oxidative Medicine and Cellular Longevity*.

[8] Kiang J. G., Fukumoto R. (2014). Ciprofloxacin increases survival after ionizing irradiation combined injury: *γ*-H2AX formation, cytokine/chemokine, and red blood cells. *Health Physics*.

[9] Ledney G. D., Elliott T. B. (2010). Combined injury: factors with potential to impact radiation dose assessments. *Health Physics*.

[10] Ledney G. D., Elliott T. B., Moore M. M., Mossman K. L., Mills W. A. (1992). Modulations of mortality by tissue trauma and sepsis in mice after radiation injury. *The Biological Basis of Radiation Protection Practice*.

[11] Ledney G. D., Stewart D. A., Exum E. D., Sheehy P. A. (1981). Skin wound-enhanced survival and myelocytopoiesis in mice after whole-body irradiation. *Acta Oncologica*.

[12] Palmer J. L., Deburghgraeve C. R., Bird M. D., Hauer-Jensen M., Kovacs E. J. (2011). Development of a combined radiation and burn injury model. *Journal of Burn Care and Research*.

[13] Alpen E. L., Sheline G. E. (1954). The combined effects of thermal burns and whole body x-irradiation on survival time and mortality. *Annuls of Surgery*.

[14] Shah K. G., Wu R., Jacob A., Blau S. A., Ji Y., Dong W., Marini C. P., Ravikumar T. S., Coppa G. F., Wang P. (2009). Human ghrelin ameliorates organ injury and improves survival after radiation injury combined with severe sepsis. *Molecular Medicine*.

[15] Valeriote F. A., Baker D. G. (1964). The combined effects of thermal trauma and x-irradiation on early mortality. *Radiation Research*.

[16] Korlof B. (1956). Infection of burns, I. A bacteriological and clinical study of 99 cases. II. Animal experiments: burns and total body x-irradiation. *Acta Chiropractic Scandinavian Supplement*.

[17] Brooks J. W., Evans E. I., Ham W. T., Reid J. D. (1952). The influence of external body radiation on mortality from thermal burns. *Annals of Surgery*.

[18] Reid J. D., Brooks J. W., Ham W. T., Evans E. I. (1955). The influence of X-radiation on mortality following thermal flash burns: the site of tissue injury as a factor determining the type of invading bacteria. *Annals of Surgery*.

[19] Baxter H., Drummond J. A., Stephens-Newsham L. G., Randall R. G. (1953). Studies on acute total body irradiation in animals. I. Effect of streptomycin following exposure to a thermal burn and irradiation. *Plastic Reconstruction Surgery*.

[20] Fukumoto R., Burns T. M., Kiang J. G. (2014). Ciprofloxacin enhances stress erythropoiesis in spleen and increases survival after whole-body irradiation combined with skin-wound trauma. *PLoS ONE*.

[21] Lausevic Z., Lausevic M., Trbojevic-Stankovic J., Krstic S., Stojimirovic B. (2008). Predicting multiple organ failure in patients with severe trauma. *Canadian Journal of Surgery*.

[22] Zou Z., Sun H., Su Y., Cheng T., Luo C. (2008). Progress in research on radiation combined injury in China. *Radiation Research*.

[23] Kiang J. G., Garrison B. R., Gorbunov N. V. (2010). Radiation combined injury: DNA damage, apoptosis, and autophagy. *Adaptive Medicine*.

[24] Kojima M., Hosoda H., Date Y., Nakazato M., Matsuo H., Kangawa K. (1999). Ghrelin is a growth-hormone-releasing acylated peptide from stomach. *Nature*.

[25] Inui A., Asakawa A., Bowers C. Y., Mantovani G., Laviano A., Meguid M. M., Fujimiya M. (2004). Ghrelin, appetite, and gastric motility: the emerging role of the stomach as an endocrine organ. *The FASEB Journal*.

[26] Baatar D., Patel K., Taub D. D. (2011). The effects of ghrelin on inflammation and the immune system. *Molecular and Cellular Endocrinology*.

[27] Kabil N. N., Seddiek H. A., Yassin N. A., Gamal-Eldin M. M. (2014). Effect of ghrelin on chronic liver injury and fibrogenesis in male rats: possible role of nitric oxide. *Peptides*.

[28] Xu X., Jhun B. S., Ha C. H., Jin Z.-G. (2008). Molecular mechanisms of ghrelin-mediated endothelial nitric oxide synthase activation. *Endocrinology*.

[29] Chen X., Chen Q., Wang L., Li G. (2013). Ghrelin induces cell migration through GHSR1a-mediated PI3K/Akt/eNOS/NO signaling pathway in endothelial progenitor cells. *Metabolism: Clinical and Experimental*.

[30] Peng Z., Zhu Y., Zhang Y., Wilhelmsen K., Jia C., Jin J., Xue Q., Feng X., Zhang F., Yu B. (2012). Effects of ghrelin on pulmonary NOD2 mRNA expression and NF-*κ*B activation when protects against acute lung injury in rats challenged with cecal ligation and puncture. *International Immunopharmacology*.

[31] Ogura Y., Inohara N., Benito A., Chen F. F., Yamaoka S., Núñez G. (2001). Nod2, a Nod1/Apaf-1 Family Member That Is Restricted to Monocytes and Activates NF-κB. *Journal of Biological Chemistry*.

[32] Dixit V. D., Yang H., Cooper-Jenkins A., Giri B. B., Patel K., Taub D. D. (2009). Reduction of T cell-derived ghrelin enhances proinflammatory cytokine expression: implications for age-associated increases in inflammation. *Blood*.

[33] Montgomery C. A. (1990). Oncologic and toxicologic research: alleviation and control of pain and distress in laboratory animals. *Cancer Bulletin*.

[34] Tomasivic S. P., Coghlan L. G., Gray K. N., Mastromarino A. J., Travis E. L. (1988). IACUC evaluation of experiments requiring death as an end point: a cancer center's recommendations. *Laboratory Animals*.

[35] International Standardization Organization and ASTM International (2013). *Standard Practice for Use of an Alanine-EPR Dosimetry System*.

[36] Wu P., Nelson E. A., Reid W. H., Ruckley C. V., Gaylor J. D. S. (1996). Water vapour transmission rates in burns and chronic leg ulcers: Influence of wound dressings and comparison with in vitro evaluation. *Biomaterials*.

[37] Tong J., Dave N., Mugundu G. M., Davis H. W., Gaylinn B. D., Thorner M. O., Tschöp M. H., D'Alessio D., Desai P. B. (2013). The pharmacokinetics of acyl, des-acyl, and total ghrelin in healthy human subjects. *European Journal of Endocrinology*.

[38] Akamizu T., Takaya K., Irako T., Hosoda H., Teramukai S., Matsuyama A., Tada H., Miura K., Shimizu A., Fukushima M., Yokode M., Tanaka K., Kangawa K. (2004). Pharmacokinetics, safety, and endocrine and appetite effects of ghrelin administration in young healthy subjects. *European Journal of Endocrinology*.

[39] Jacob A., Shah K. G., Wu R., Wang P. (2010). Ghrelin as a novel therapy for radiation combined injury. *Molecular Medicine*.

[40] Cheyuo C., Wu R., Zhou M., Jacob A., Coppa G., Wang P. (2011). Ghrelin suppresses inflammation and neuronal nitric oxide synthase in focal cerebral ischemia via the vagus nerve. *Shock*.

[41] Stoyanova I. I. (2011). Ghrelin: expression and functions in the central nervous system. *Ghrelin: Production, Action Mechanisms and Physiological Effects*.

[42] Metcalf D., Neu H. C. (1990). The role of the colony-stimulating factors in the treatment of infections. *Frontiers of Infectious Diseases: New Antibacterial Strategies*.

[43] Metcalf D. (2008). Hematopoietic cytokines. *Blood*.

[44] Badiavas E. V., Abedi M., Butmarc J., Falanga V., Quesenberry P. (2003). Participation of bone marrow derived cells in cutaneous wound healing. *Journal of Cellular Physiology*.

[45] Stevanovic D., Starcevic V., Vilimanovich U., Nesic D., Vucicevic L., Misirkic M., Janjetovic K., Savic E., Popadic D., Sudar E., Micic D., Sumarac-Dumanovic M., Trajkovic V. (2012). Immunomodulatory actions of central ghrelin in diet-induced energy imbalance. *Brain, Behavior, and Immunity*.

[46] Chen T., Burke K. A., Zhan Y., Wang X., Shibata D., Zhao Y. (2007). IL-12 facilitates both the recovery of endogenous hematopoiesis and the engraftment of stem cells after ionizing radiation. *Experimental Hematology*.

[47] Xia Q., Pang W., Pan H., Zheng Y., Kang J.-S., Zhu S.-G. (2004). Effects of ghrelin on the proliferation and secretion of splenic T lymphocytes in mice. *Regulatory Peptides*.

